# Commissioning of the world's first compact pencil‐beam scanning proton therapy system

**DOI:** 10.1002/acm2.12225

**Published:** 2017-11-20

**Authors:** Rajesh Pidikiti, Bijal C. Patel, Matthew R. Maynard, Joseph P. Dugas, Joseph Syh, Narayan Sahoo, Hsinshun Terry Wu, Lane R. Rosen

**Affiliations:** ^1^ Procure Proton Therapy Center Oklahoma City OK USA; ^2^ Radiation Oncology Methodist Hospital Baytown TX USA; ^3^ Radiation Oncology Willis‐Knighton Cancer Center Shreveport LA USA; ^4^ Division of Radiation Oncology Department of Radiation Physics The University of Texas MD Anderson Cancer Center Houston TX USA

**Keywords:** clinical commissioning, PBS, pencil beam, Proteus^®^ONE, proton, radiotherapy, spot scanning

## Abstract

This paper summarizes clinical commissioning of the world's first commercial, clinically utilized installation of a compact, image‐guided, pencil‐beam scanning, intensity‐modulated proton therapy system, the IBA Proteus^®^ ONE, at the Willis‐Knighton Cancer Center (WKCC) in Shreveport, LA. The Proteus^®^ ONE is a single‐room, compact‐gantry system employing a cyclotron‐generated proton beam with image guidance via cone‐beam CT as well as stereoscopic orthogonal and oblique planar kV imaging. Coupling 220° of gantry rotation with a 6D robotic couch capable of in plane patient rotations of over 180° degrees allows for 360° of treatment access. Along with general machine characterization, system commissioning required: (a) characterization and calibration of the proton beam, (b) treatment planning system commissioning including CT‐to‐density curve determination, (c) image guidance system commissioning, and (d) safety verification (interlocks and radiation survey). System readiness for patient treatment was validated by irradiating calibration TLDs as well as prostate, head, and lung phantoms from the Imaging and Radiation Oncology Core (IROC), Houston. These results confirmed safe and accurate machine functionality suitable for patient treatment. WKCC also successfully completed an on‐site dosimetry review by an independent team of IROC physicists that corroborated accurate Proteus^®^ ONE dosimetry.

## 1 INTRODUCTION

Proton therapy facilities are traditionally large, both in physical size and in requisite staffing for effective operation, often making them prohibitively expensive for community or regional cancer centers. Traditional passive scattering and uniform scanning systems can also contribute unwanted neutron dose, a byproduct of proton interaction with beam‐shaping components, to healthy tissue. Recent advances in proton therapy technology are changing both of these traditional standards with movement toward pencil beam‐scanning (PBS) systems and development of smaller and of less expensive single‐room compact proton therapy systems. PBS systems offer both improved target dose conformity and reduced neutron dose as compared to uniform scanning systems because they are capable of delivering both single‐field uniform dose and multiple‐field intensity modulated proton therapy without the need for compensators or apertures. Scanning beam systems have been developed by Hitachi, IBA, and other companies^.^ 1, 2, 3

At the Willis‐Knighton Cancer Center (WKCC) in Shreveport, LA, the world's first commercial, compact, image‐guided, pencil‐beam scanning proton therapy system, the IBA Proteus^®^ONE, has been installed and commissioned. The system began treating patients in September 2014 and to‐date has treated more than three hundred and thirty patients. The Proteus^®^ONE offered easy integration into Willis‐Knighton's established conventional therapy center in terms of space and cost while providing physicians added flexibility in choosing the best modality for case‐specific treatment. Proton treatment planning at WKCC is performed using the RayStation treatment planning system (TPS) (RaySearch Laboratories, Stockholm, Sweden), while the oncology information system (OIS) used is MOSAIQ (IMPAC Medical Systems, Inc., Sunnyvale, CA, USA).

Along with general machine characterization, this paper describes the clinical commissioning of the IBA Proteus^®^ONE undertaken to ensure both safe and accurate patient treatment. System commissioning required: (a) characterization and calibration of the proton beam, (b) TPS commissioning including CT‐to‐density curve determination, (c) image guidance system commissioning, and (d) safety verification (interlocks and radiation survey).

## 2 METHODS AND MATERIALS

### 2.A Proteus^®^ONE overview

**Figure 1:**
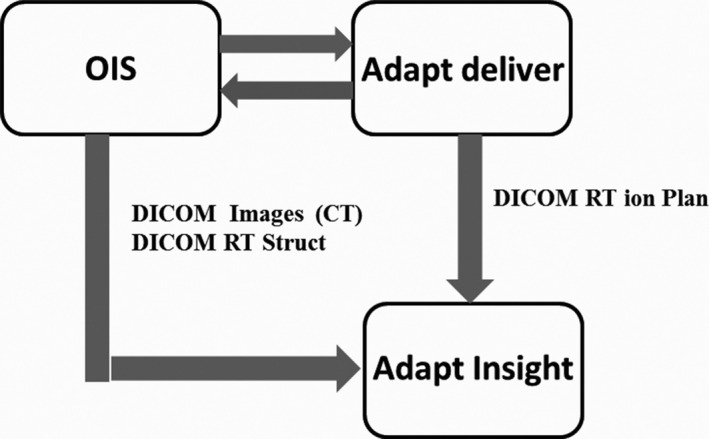
Data interface for patient treatment with a continous spot scanning proton beam using the Proteus^®^ ONE system.

The IBA Proteus^®^ONE features an isochronous cyclotron, 220° partial‐rotation compact gantry, scanning beam delivery nozzle, image guidance system with cone‐beam CT and stereoscopic imaging capabilities, and a 6D robotic couch. Continuous dynamic spot scanning of the cyclotron‐generated proton beam, coupled with rapid adjustment of beam energy, is used to treat three dimensional target volumes. Discrete dose deposition layers within a target, ranging from surface to 32‐cm water equivalent thickness (WET), are achieved via adjustment of beam energy by a degrader and energy selection system (ESS) located between the cyclotron and PBS delivery nozzle. Within each layer, scanning magnets direct discrete beamlets (spots) along the x‐ and y‐directions yielding a maximum proton field size of 20 × 24 cm in x‐ and y‐directions at isocenter. Although IBA Proteus^®^ONE hardware produces and supplies the proton beam, additional equipment from both IBA and other vendors is necessary to achieve patient treatment delivery. A third‐party TPS computes required layer energies, along with spot locations and weights, based on patient morphology for a set of user‐specified beam (gantry) and couch angles. These patient‐specific plan parameters, along with reference image datasets, are then transferred to a third‐party OIS, which is also used to transfer both plans and image datasets to IBA's adaPTdeliver^TM^ console system and adaPTinsight^TM^ imaging system software, respectively, for treatment delivery as shown in Fig. 1. Accurate, seamless integration between these disparate systems is a crucial piece of the Proteus^®^ONE system.

#### 2.A.1 Accelerator

Unique to Willis‐Knighton's Proteus^®^ONE installation is the use of an IBA C230 cyclotron, which is to be replaced with the IBA S2C2 super‐conducting cyclotron at subsequent Proteus^®^ONE sites, to accelerate the protons. The C230 cyclotron is configured to produce a 230 MeV beam with an accelerating voltage frequency of 106.1 MHz. The range of extracted current is 1 to 300 nA with an extraction efficiency of 60% ± 10%. The ion source's turn on/off time is 15 μs with a 45 μs transit time from ion source to patient. Range is modulated via an energy degrader composed of variable block thicknesses of beryllium, graphite, and aluminum that allows for beam energies of 70 MeV (4.1 g/cm^2^ water) to 230 MeV (32.95 g/cm^2^ water). Measurement of revolution frequency and orbit position during beam extraction from the cyclotron verifies proton energy. IBA determined that the beam orbit position within the cyclotron is within ±1 mm of optimal, assuring proton range accuracy to within 0.025 g/cm^2^.

#### 2.A.2 IBA‐PBS delivery nozzle

**Figure 2:**
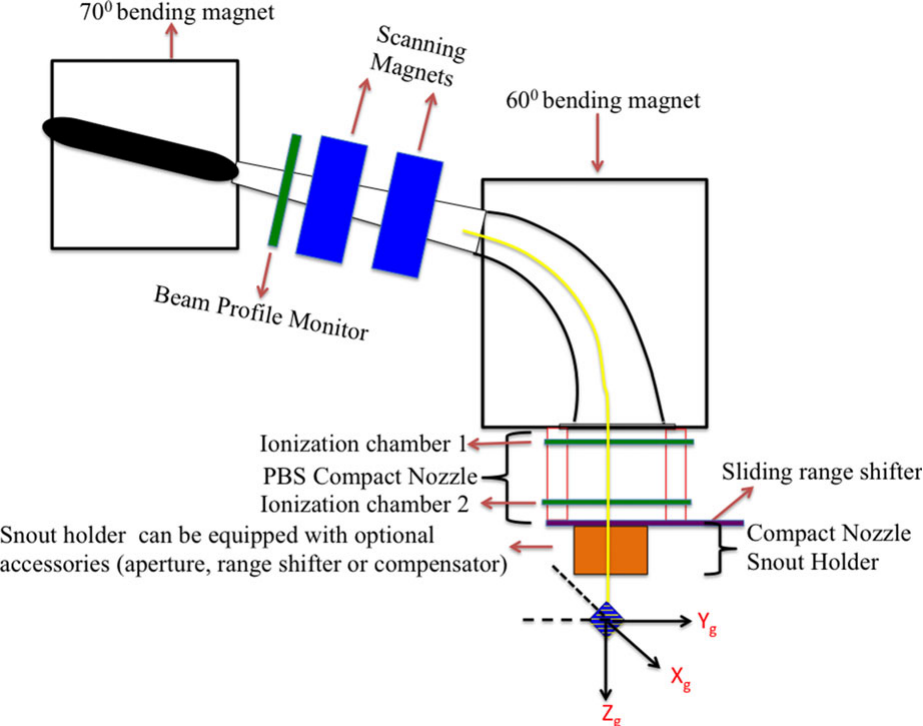
A schematic representation of the Proteus^®^ ONE's PBS compact nozzle beam delivery system.

Specifically designed for pencil beam scanning delivery, Proteus^®^ONE's PBS nozzle is diagramed in Fig. 2. A beam profile monitor, located downstream of the 70° bending magnet, checks beam‐specific parameters such as spot size and symmetry. Scanning magnets, located within the nozzle between the 70° and 60° bending magnets, change the pencil beam position based on the treatment plan with settings taken from a lookup table. A scanning controller ensures that beam energy, spot position, and MUs (dose) per spot are delivered as specified by the treatment plan with beam pauses between each spot delivery. Spot‐to‐spot delivery time within a layer is on the order of milliseconds, while energy layer switching time is approximately 0.9 s. The final 60° bending magnet (wide‐gap dipole) directs the scanned beam toward the final components of the delivery nozzle, known as the PBS compact nozzle and compact nozzle snout holder, respectively, and the patient.

The PBS compact nozzle contains two ionization chambers, a compact nozzle snout holder (CNSH), and a sliding accessory drawer. The ionization chambers (IC1, IC2) are open design and require corrections for temperature and pressure to be manually input every 14 hr. Each IC is composed of multiple layers that work in tandem to verify dose output, spot size, and alignment (spot position). An integral dose plane collects charge generated by the incident proton beam to verify delivered MU (i.e., absolute dose per spot), while strips planes composed of parallel wires acquire what are essentially 1D profiles of the incident proton beamlet to verify spot position, symmetry and skewness. Minimum and maximum MUs per spot are limited to 0.01–12 MU by the control software, values that correspond to the limits of IC2 accuracy, for treatment accuracy and patient safety. Tolerance for each spot position is 1 mm or ±10% beam sigma of the requested x‐ and y‐positions with values outside of tolerance causing the scanning controller system to pause treatment. The accessory drawer can be used to support patient specific apertures, range compensators, and/or range shifters (energy absorbers) that may be required by the treatment plan. The drawer can translate into and out of the beamline, as well as along the beam path toward or away from the patient.

Two Lexan range shifters with WETs of 4.1 and 7.4 cm (physical thicknesses of 3.5 and 6.5 cm, respectively) were provided by IBA. Interposing a range shifter into the beam reduces beam energy, allowing treatment of superficial targets and full dose modulation up to the skin surface with the 4.1‐cm device corresponding to the range of 70 MeV protons and the 7.4‐cm device corresponding to that of 100 MeV protons. Although commissioning measurements for both range shifters were acquired, WKCC saw little practical need for the 7.4 cm device as the Proteus^®^ONE is capable of producing 70 MeV protons. Furthermore, our group has shown that the 7.4 cm range shifter has, on average, 50% greater TPS dosimetric error than its 4.1 cm counterpart.^4^ Therefore, only the 4.1‐cm range shifter is clinically commissioned with its effects on spot size considered in the beam modeling process.

In addition to the range shifters, circular Proteus^®^ONE snouts were provided in two diameters: 24.4 and 10.6 cm. These snouts, which are physical attachments that can be fitted on to the compact nozzle, would typically be used to place an additional range shifter and aperture closer to complex patient anatomy. However, because these optional attachments were not yet supported by the TPS at the time of commissioning, they were not included in the commissioning process. Currently, Proteus^®^ONE snouts are not clinically utilized at WKCC.

### 2.B Characterization and calibration of the proton beam

Beam characterization included but was not limited to TPS mandates for beam modeling. The RayStation TPS required in‐air spot profiles, integrated depth doses (IDD), and absolute dose per monitor unit measured at a depth between 1 cm and one‐half of the Bragg peak maximum.

#### 2.B.1 Beam measurements

Utilizing an IBA Blue Phantom^2^, pristine Bragg peak beams were measured in water using a large‐area Bragg peak (BP) chamber (PTW‐Freiburg Model 3407) in 5 MeV increments from 70 to 226.7 MeV. The BP chamber's large size (10.5‐cc nominal sensitive volume, 4.2‐cm effective radius) captures charge generated by the beam spot, including scattered protons, providing an integrated depth dose (IDD) measurement. IDDs were measured over the entire range of each pristine Bragg peak with 1 mm spacing in the upward sweep region and 0.5 mm spacing in the Bragg peak region. For all beam energies, the measured R_90_ was compared to IBA's predicted value, calculated using an energy to range conversion based on ICRU 49.^5^ Other parameters including minimum range, maximum range, and WET of the range shifter were evaluated for a few energies.

Single spot profiles were measured in both air and RW3 solid water (characterization only) at zero‐degree gantry angle using a scintillator‐based Lynx detector (IBA Dosimetry Schwarzenbruck, Germany). Lynx workflow was documented by Lin et al.,^6^ with its suitability for proton therapy beam quality assurance checks reported by Lin et al.^7^, ^8^ Spot profiles were acquired in 5 MeV energy steps from 70 to 226.7 MeV at distances of +16, ‐16, and 0 cm along the beam line from isocenter. Spot profiles were also measured in air with the range shifter in place for three energies (115, 170, and 226.7 MeV), again at distances of +16, ‐16, and 0 cm along the beam line from isocenter. Spot profiles measured at several locations along the beam path allowed Raysearch to quantitate beam divergence.

A series of simple spot positioning tests were also performed to verify spot placement accuracy. For the clinical range of beam energies, spots were placed ±5 and ±10 cm from the origin along the *x*‐axis and ±6 and ±12 cm from the origin along the *y*‐axis, and measured using the Lynx to quantitate the distances between the spots. This process was then repeated with spots placed along the diagonal.

#### 2.B.2 Absolute dose calibration

For passive scattering proton therapy systems, the relationship between absolute dose and monitor units can be established by the IAEA TRS398 protocol.^9^ However, TRS398 does not adequately cover the dosimetry of PBS systems.^10^, ^11^ As such, a pencil beam scanning system requires a different setup for absolute dose per MU calibration. As there is, initially, no beam model for the TPS to use to create an SOBP, 32 individual, single‐energy scanned fields were created as PLD (PBS Layers Definition) files, an IBA‐specific format which allows the system to deliver pristine layers independently from the OIS. To fulfill RayStation beam modeling requirements, each PLD was defined with a field size of 10 × 10 cm^2^ and 2.5‐mm spot spacing (grid size of 1681 = 41 × 41 spots). These single energy fields were created in increments of 5 MeV from 70 to 226.7 MeV, each with 1 MU per spot (1681 total MU) to create a uniform lateral profile. Doses were measured using a PPC05 parallel plate chamber and a UNIDOS electrometer, both calibrated by an Accredited Calibration Laboratory (ADCL). The chamber was placed at 2.0 cm depth in water including the water equivalent thickness of the chamber window. Chamber readings were corrected for temperature and pressure, with P_ion_ and P_pol_ corrections calculated using the two‐voltage method and assuming a continuous beam. P_ion_ and P_pol_ corrections tracked with beam energy and accounted for maximum and minimum dose differences of 0.4% (226.7 MeV) and 0.09% (70 MeV). The magnitudes of these corrections were in line with data reported by Gao.^12^ Results were scaled with RBE (Relative Biological Effectiveness) of 1.1 to match that used in the TPS. This same setup was used for three different energies (130, 170, and 226.7 MeV) measured with the range shifter in place. These absolute doses per MU measurements can be combined with relative depth dose curves to provide dose at any depth along the beam path.

As discussed in Section 2.C., all necessary data were sent to RaySearch for beam modeling. The resultant beam model was validated by generating a deliverable treatment plan and delivered a uniform dose of 200 cGy to a cube of 10 × 10 × 10 cm^3^. A total of 1550 MUs were specified by the plan corresponding to energies of 120 to 180 MeV (proton ranges of 10.1–20.1 g/cm^2^) to produce a uniform 10 cm SOBP. To verify monitor units, absolute dose measurement was performed using a waterproof, ADCL‐calibrated PPC05 chamber placed at a reference depth of 15 g/cm^2^. MU linearity of the nozzle's ionization chambers was also verified by scaling the plan's MUs to deliver 0.6, 0.8, 2, and 5 times the original dose.

#### 2.B.3 Variable virtual SAD (VSAD) measurement

The unique orientation of the scanning and bending magnets of the Proteus^®^ONE compact gantry causes the virtual source‐to‐axis distance to vary along the direction of the bending magnet (cranio‐caudal with head‐first supine patient position and treatment couch at 0°). A Lynx PT scintillator‐based sensor was used to verify the magnitude and range of the VSAD via measurement of a pattern of spots (one central and two at each max deflection) at three vertical positions: isocenter, 16 cm from isocenter toward the nozzle, and 16 cm from isocenter away from the nozzle. Values for the VSAD along the bending magnet direction were then determined geometrically.

To evaluate clinical impact, Willis‐Knighton collaborated with IBA and RaySearch to implement a simple variable VSAD model in RayStation. While the actual trend of VSAD is slightly parabolic, a linear fit of the measured data was adopted to approximate its effect in the TPS. Phantom treatment plans were generated at clinically relevant treatment depths for various sites (e.g., prostate, whole pelvis, and cranium). Calculated dose distributions with and without VSAD consideration were compared for each plan.

### 2.C TPS commissioning

#### 2.C.1 Raystation beam modeling

Beam modeling for the TPS required in‐air spot profiles, integrated depth doses (IDD), and absolute dose measured at a depth between 1 cm and one‐half of the Bragg peak maximum. While RaySearch recommends these measurements be taken at a depth equal to the midpoint of this range, for simplicity, and with input from RaySearch, we performed all absolute dose measurements at a depth of 2 cm from the water surface for each mono‐energetic beam. Beam data obtained with the range shifter in place was not required by RayStation as its effect is modeled within the dose engine. All necessary beam data were supplied to RaySearch Laboratories who then generated the final beam model, the details of which are beyond the scope of this paper.

#### 2.C.2 CT‐to‐density curve determination

Our clinic utilizes a Phillips Brilliance Big Bore 16‐slice CT scanner for patient simulation. Using Gammex 467 tissue characterization plugs inside acrylic phantoms, we studied various CT acquisition settings and phantom sizes to establish imaging protocols and create a CT‐to‐density table from which the TPS estimates relative stopping power ratios for proton beam dose calculations.^5^, ^13^, ^14^ These protocols were tested in the TPS by comparing the mass densities determined by the protocols in patient CT datasets against reference ICRU 49 data for known human tissues.^5^

Accurate dose calculation in proton therapy depends on proton relative stopping power ratios. RayStation uses an internal mass density to stopping power conversion during dose calculation. Individual CT voxels are assigned a known biological material based on mass density. The stopping power is then calculated on the fly using the density of the voxel, the properties of the known material (e.g., mean excitation potential and elemental composition) and the Bethe‐Bloch equation. A stoichiometric calibration was also independently performed by an external proton physicist to verify stopping powers calculated by RayStation.

#### 2.C.3 TPS validation

Measurement of spot profiles in solid water, depth doses for inversely optimized plans, lateral dose profiles, dose uniformity, absolute dose, and patient treatment field specific QA in homogenous phantoms were used to both verify the new beam model and validate the TPS. Twenty‐three different treatment plans generating cube patterns of uniform dose over the SOBP for varying field sizes, ranges (depths), and prescribed doses were produced in RayStation version 4.7. SOBPs ranged from 2 to 10 cm in depth for field sizes of 4 to 18 cm and spot spacings of 3 to 8 mm. Isocenter location varied from 8 to 26 cm in depth. Air gaps ranged from 5.4 to 25 cm for range shifter plans. Dose measurements of these plans were performed using a PPC05 ionization chamber both on and off central axis, with and without a range shifter. Absolute and relative gamma analyses were performed, respectively, between MatriXX^PT^‐measured and Lynx^PT^‐measured planar dose profiles and TPS‐calculated planar doses.

Pristine Bragg peaks and SOBPs were measured using the Zebra^TM^ (IBA Dosimetry, USA), multi‐layer ion chamber device for energies ranging from 6 to 32 g/cm^2^ water equivalent depth. Depth‐dose characteristics such as peak width and peak position were compared against their TPS counterparts. Similarly, spot measurements in solid water were verified by comparing Lynx measurements against TPS‐calculated profiles. End‐to‐end tests covered simulation, contouring, treatment planning, plan review, MU measurement, QA, and plan delivery. QA plans were created in the TPS to compare against measurements made using an IBA MatriXX^PT^ ionization chamber array. Anthropomorphic phantom irradiations (IROC prostate, lung, head and neck) were used as final dose verification. A breath‐hold technique was used in the lung phantom to minimize the target motion.

### 2.D Image guidance system commissioning

#### 2.D.1 Image guidance system overview

The Proteus^®^ONE has two independent systems that can be used for three modes of image guidance. One is a fixed kV‐kV stereoscopic system consisting of two floor‐mounted x‐ray tubes, positioned 60^0^ relative to each other and 45^0^ relative to the floor, with two ceiling‐mounted flat panel detectors. The second consists of a gantry‐mounted retractable x‐ray tube and a retractable image panel, which can be used to acquire planar radiographs or to perform cone beam CT. The adaPTinsight^TM^ software application coupled with the Proteus^®^ONE's 6D robotic couch provides a streamlined, single‐user interface for proton therapy patient positioning and treatment delivery enabling the user to perform image‐guided proton therapy (IGPT). In all three modes (kV‐kV planar, stereoscopic, or CBCT) the software allows for either manual or automatic 6D registration of the acquired images against a reference dataset. Image registration is based on mutual information between acquired and reference images and a correction vector is computed that can be applied via a virtual hand pendant on the adaPTinsight^TM^ control station. During commissioning, standard tests of x‐ray parameters and imaging quality, which will not be detailed herein, were performed for all modalities. Couch isocentricity and table sag were also measured and verified with each imaging system.

#### 2.D.2 Stereoscopic system characterization

Of primary concern during stereoscopic system characterization were both geometric accuracy and geometric integrity between the proton beam axis and stereoscopic imaging system axis. Geometric accuracy was evaluated by imaging a 5‐cm diameter steel ball placed at isocenter using both detector panels and measuring the imaged ball diameter across multiple directions ranging from 0 to 350 degrees. Coincidence between radiation and stereoscopic isocenters was evaluated with radiochromic film and a scintillator‐based detector. Using the stereoscopic imaging system, the steel ball was aligned at isocenter to within 0.2 mm as determined via isocenter comparisons on each oblique radiograph. A radiochromic film was then placed downstream of the ball as near as possible to isocenter and a circular spot pattern delivered. Analysis of the resulting concentric circles on the film was performed, with the Euclidian distance between the centers of both circles indicating the degree of radiation and stereoscopic collinearity. This procedure, including realignment of the steel ball with stereoscopic imaging, was repeated for multiple gantry angles and beam energies.

Isocentricity was also verified with the XRV100 scintillator (Logo System Intl, CA, USA),^7^ which works on the principle of a hodoscope and can measure beam isocenter based on particle trajectory through the surface of a scintillator cone. The XRV100 was imaged with the CT scanner to create a reference image set, which was then transferred to adaPTinsight^TM^. The XRV100 was aligned in the treatment room by iteratively performing stereoscopic imaging and registration until residual corrections vectors were minimal. Isocentricity was then confirmed by exposing the XRV100 to single spot pristine beams from multiple gantry angles for a range of energies.

#### 2.D.3 Gantry mounted imaging system

The confirmation of radiation isocenter and stereoscopic imaging isocenter collinearity provided the ability to verify the gantry‐mounted imaging system (kV‐kV orthogonal radiographs and cone‐beam CT) against the stereoscopic imaging system. A cube phantom with a central BB marker was first aligned to isocenter using stereoscopic oblique radiographs. Orthogonal kV‐kV radiographs and both clockwise (CW) and counter clockwise (CCW) CBCTs of the cube phantom were then obtained and their respective registration shifts evaluated to confirm isocenter agreement between the stereoscopic and gantry‐mounted imaging systems.

## 3 RESULTS

### 3.A Beam characterization

**Figure 3:**
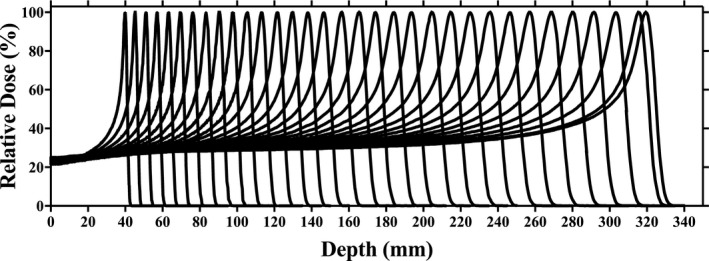
Integrated depth doses (individually normalized to 100%) acquired using a Bragg Peak chamber for energies of 70–226.7 MeV.

**Figure 4:**
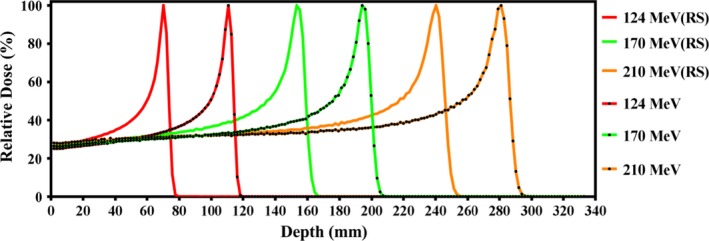
Normalized Pristine Bragg peaks measured in water using a Bragg Peak chamber with and without a range shifter (RS) in place.

IDD measurements with the Bragg peak chamber for energies of 70–226.7 MeV in increments of 5 MeV are shown in Fig. 3. Data are normalized to 100% at maximum dose. The range is defined by 90% on the distal edge of the Bragg peak. Table 1 compares measured R_90_ against the IBA‐predicted range based on ICRU 49 data. All energies exhibited good agreement between measured and predicted ranges. The IDD measurements were less than ±0.15 g/cm^2^ at the distal fall‐off as compared with ICRU 49 data. Pullback of the pristine Bragg peak by the 4.1 cm (WET) range shifter was evaluated for energies of 124, 170, and 210 MeV as shown in Fig. 4. The water equivalent depth for the range shifter was evaluated with Bragg peak measurements and the result was within 0.1 g/cm^2^.

**Table 1:** Measured (Zebra) and expected R_90_ for several pristine beams

Energy [MeV]	Measured R_90_ [cm]	Expected R_90_ [cm]	Difference [mm]
70	4.04	4.08	0.4
75	4.58	4.62	0.4
80	5.17	5.18	0.1
85	5.78	5.78	0.0
90	6.4	6.4	0.0
95	7.05	7.05	0.0
100	7.73	7.72	−0.1
105	8.46	8.42	−0.4
110	9.15	9.14	−0.1
115	9.89	9.89	0.0
120	10.67	10.66	−0.1
125	11.46	11.46	0.0
130	12.3	12.28	−0.2
135	13.13	13.12	−0.1
140	13.96	13.99	0.3
145	14.82	14.87	0.5
150	15.79	15.78	−0.1
155	16.74	16.71	−0.3
160	17.59	17.66	0.7
165	18.62	18.63	0.1
170	19.56	19.62	0.6
175	20.68	20.63	−0.5
180	21.69	21.66	−0.3
185	22.76	22.71	−0.5
190	23.81	23.77	−0.4
195	24.86	24.86	0.0
200	25.95	25.96	0.1
205	27.12	27.09	−0.3
210	28.26	28.23	−0.3
215	29.4	29.38	−0.2
220	30.57	30.56	−0.1
225	31.79	31.75	−0.4
226.7	32.18	32.16	−0.2

**Figure 5:**
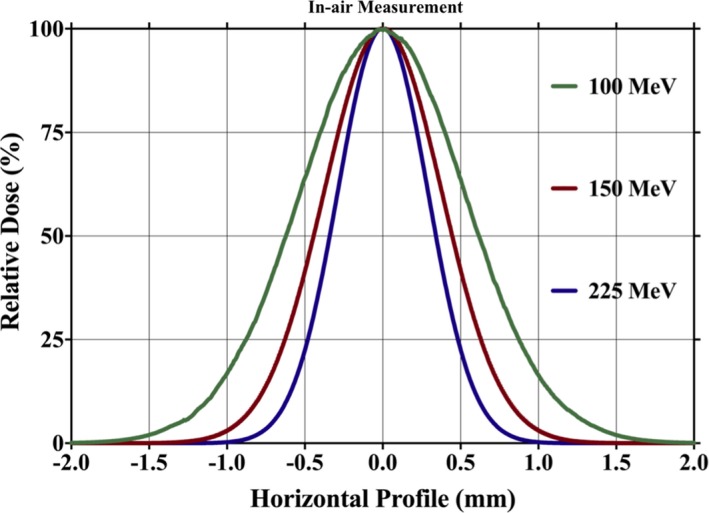
Spot profiles in air at isocenter for three different energies: 100, 150, and 225 MeV.

The spot profiles in Fig. 5 show Gaussian beam distribution measured in air for three different energies: 100, 150, and 225 MeV. The difference between measured and calculated sigma‐x and sigma‐y values in air was found to be within tolerance of 0.2 mm. The spot position and characteristics along the central ray were compared to the spots located at 12 cm along the y‐direction and 10 cm along x‐directions. These spot position checks were performed at various gantry angles and the deviations from the planned positions were found to be less than 1 mm.

**Figure 6:**
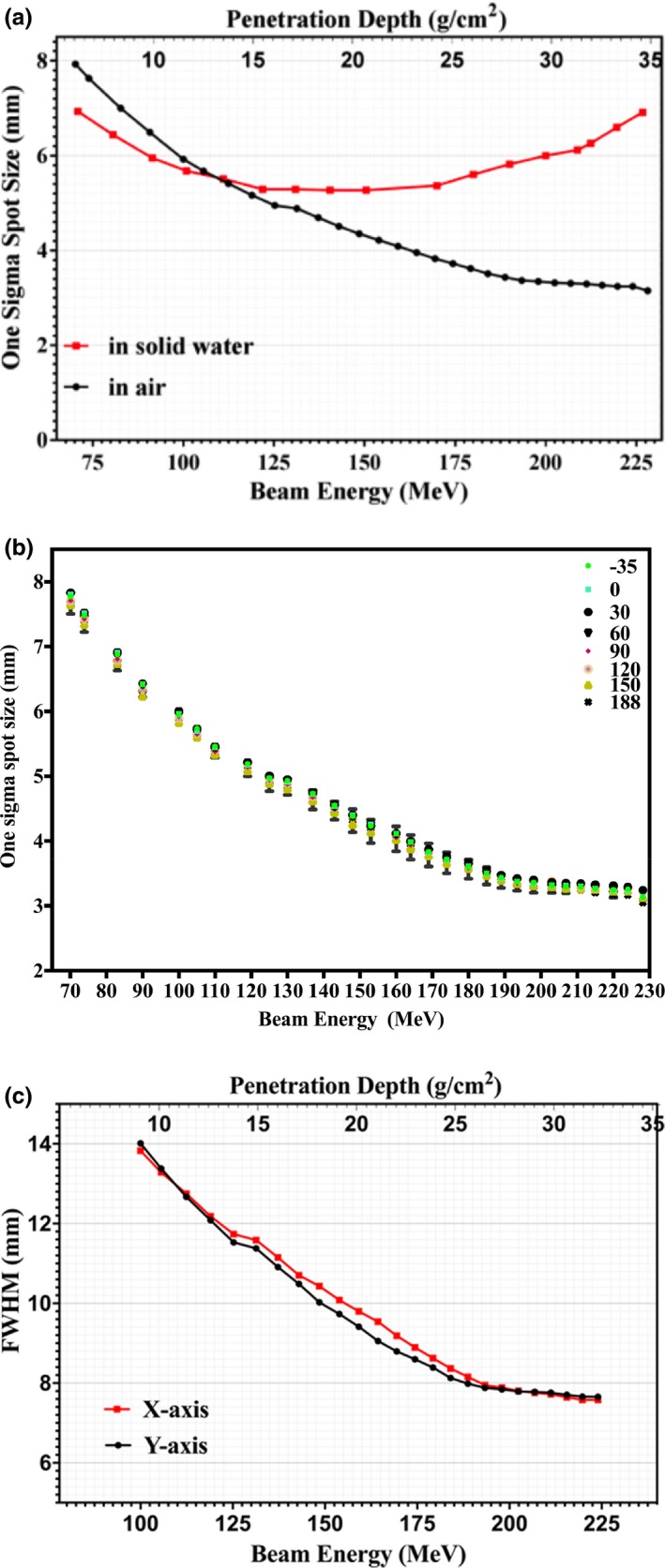
(a) One sigma spot size of profiles in air and in solid water of single pencil beams at the isocenter plane as function of beam energy and depth. (b) One sigma spot size of profiles in air single pencil beams at the isocenter plane as function of depth and gantry angle. (c) FWHM of lateral profiles in air of single pencil beams at the isocenter plane as function of beam energy and depth.

The spot sizes in terms of one beam sigma of the single spot in water and in air are shown in Fig. 6(a) for energies of 70 to 226.7 MeV. Spot full width at half maximum (FWHM) in solid water depends on measurement depth, with larger FWHM seen at deeper depths because of multiple Coulomb scattering of protons. The effect of bending magnet strengths at various gantry angles on spot size is shown in Fig. 6(b). The variation in spot size FWHM in air along the x‐ and *y*‐axis is due to the different directions of the steering magnets in the nozzle, which causes asymmetry between in‐beam divergence and spot sigma along the x and y directions as shown in Fig. 6(c).

### 3.B Absolute dose calibration

Using the TRS398 protocol^9^ and measurement of dose at the middle of the SOBP with depth of 15 g/cm^−2^, the ratio of measured dose to TPS dose was found to be within ±0.5% for measurements inside the TPS‐calculated volume both on and off central axis. Twenty measurements performed throughout the field at various depths confirmed TPS‐predicted doses. Measured dose in the center of the volume at isocenter as compared to TPS calculation was within 0.2%. Additional plans and absolute dose measurements were performed as part of the TPS validation as described in Section 3.D.

### 3.C CT‐to‐density table determination

Five patients (three pelvis and two prostate) were scanned and evaluated for adipose, muscle, and cortical bone physical density data as compared to the ICRU 49 protocol.^5^ These density comparison results are summarized in Table 2. Relative linear stopping power ratios (RLSP) for various tissues and materials at 100 MeV were compared for several methods of calculation, including RayStation's mass density approach (HU→*ρ*→RLSP), an on‐site stoichiometric calibration (HU→RLSP) performed by an independent physicist, an additional mass density‐based method presented by Fippel and Soukup (*ρ*→RLSP),^15^ and, where applicable, ICRU 49.^5^ RLSP comparisons for adipose, muscle, and cortical bone were performed for the five patients mentioned previously and are presented in Table 2. RLSP comparisons for the Gammex tissue characterization plugs are presented in Table 3.

**Table 2:** Physical density and relative linear stopping power comparisons for three tissue types sampled from five patient CT datasets

Name	Tissue	ICRU 46 Density		RLSP‐100 MeV			
	Avg HU	ρ [g/cm^−3^]	ρ_ICRU_ [g/cm^−3^]	RayStation^a^	Stoich.^b^	Fippel^c^	ICRU 49
Adipose	−108	0.93	0.92	0.945	0.951	0.958	0.946
Muscle	47	1.07	1.04	1.053	1.047	1.064	1.028
Cortical Bone	1345	1.88	1.85	1.676	1.709	1.672	1.659

a Private communication with RaySearch Laboratories.

b Stoichiometric cal. performed on‐site by an indepdent proton physicist.

c Calculated using Eq. 13 of Fippel and Soukup.

**Table 3:** Relative linear stopping power comparisons for several Gammex tissue characterization plugs using Protocol

Gammex Material			RSLP ‐100 MeV			
Name	Avg HU	*ρ* [g/cm^−3^]	RayStation^a^	Stoich.^b^	Fippel^c^	ICRU 49
Lung 300	−726	0.29	0.287	0.287	0.287	–
Lung 450	−548	0.45	0.452	0.452	0.454	–
Adipose	−95	0.94	0.955	0.960	0.963	0.946
Breast	−46	0.99	0.990	0.993	1.001	–
Water	−5	1.00	1.000	1.000	1.000	–
Brain	21	1.05	1.034	1.029	1.047	1.0287
Liver	1	1.10	1.074	1.077	1.078	–
Inner bone	223	1.15	1.112	1.140	1.123	–
B 200 bone material	242	1.16	1.121	1.149	1.130	–
CB 2%–30% CaCO_3_	471	1.34	1.262	1.262	1.266	–
CB 2%–30% CaCO_3_	845	1.56	1.431	1.447	1.427	–
Cortical bone	1263	1.82	1.626	1.650	1.620	1.659

a Private communication with RaySearch Laboratories.

b Stoichiometric cal. performed on‐site by an indepdent proton physicist.

c Calculated using Eq. 13 of Fippel and Soukup.

### 3.D TPS validation

TPS‐calculated spot profiles were compared against measured data for a wide variety of clinical scenarios. Using the Lynx device, a total of 57 sets of measurements were obtained at various depths in solid water and proton ranges (energies), with and without the range shifter. Each measurement consisted of a layer containing 17 spots placed throughout the treatable field size (20 × 24 cm). Relative gamma analyses between measured and TPS spot profiles were all greater than 95% passing with 3%/3 mm dose/distance agreement criteria. Measured and TPS spot sigmas all agreed within 0.5 mm for both x and y directions.

The distance‐to‐agreement (DTA) between TPS‐calculated and Zebra‐calculated ranges (R_90_) was within 0.7 mm for all measured Pristine Bragg peaks. The SOBP plateau region is defined as that between the 50% point distally and the 98% proximally reduced by two distal fall‐off widths (80%–20%) on each side.

**Figure 7:**
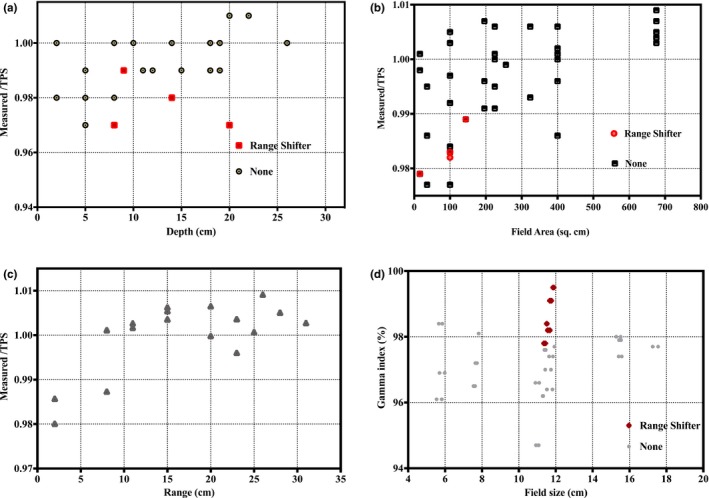
Comparison between measured and TPS calculated point doses along the central axis for various SOBP plans as a function of depth (a), field size (b), and range (c). Gamma index as a function of field size (d).

Figure 7 shows a comparison between measured and TPS‐calculated point doses along the central axis for various SOBP plans as a function of depth (a), field size (b), and range (c), along with gamma index as a function of field size (d). All point dose measurements as shown in Fig. 7 (a) through 7(c) were within 3% of the TPS calculated values, except when using a range shifter, as discussed in the next paragraph. Figure 7(d) shows that Lynx PT‐measured relative planar doses at shallow, proximal‐end, center, and distal‐end depths on the SOBP agreed well with the TPS calculated dose distribution with gamma pass rates greater than 95% for all measured field sizes when using dose/distance agreement criteria of 3% and 3 mm.

**Figure 8:**
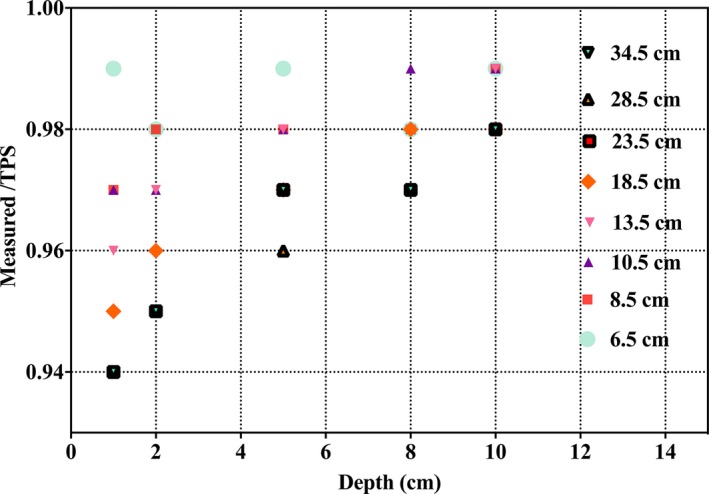
Comparison between measured and TPS‐calculated point doses at various depths as a function of the air gap between the range shifter and phantom.

**Figure 9:**
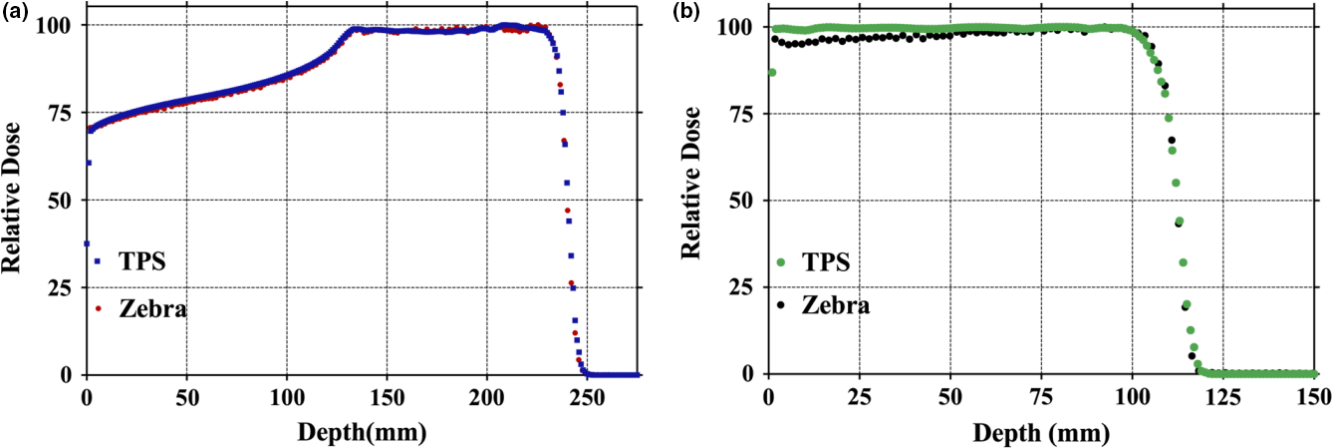
(a) Comparison between Zebra‐measured and TPS‐calculated dose distributions. (b) Comparison between Zebra‐measured and TPS‐calculated dose distributions with a range shifter.

A comparison between measured and TPS‐calculated point doses at various depths as a function of the air gap between the range shifter and phantom is given in Fig. 8. A disagreement of approximately 6% (dose overestimation by TPS) was observed for a completely retracted range shifter at a proximal measurement depth of 1.5 cm. Relative depth dose comparisons for these test plans, normalized to the maximum SOBP dose, were also compared via Zebra^TM^ measurements as shown in Fig. 9. The results yield a less than 1 mm difference at the distal 90% edge in all cases. However, as observed with absolute dose discussed above, measured relative depth dose profiles are lower than their TPS‐calculated counterparts on the proximal edge when there is a large air gap. The reason for this disagreement will be discussed in Section 4.

Final dose validation results from our IROC anthropomorphic phantom irradiations are given in Table 4. All measurements met their respective IROC passing criteria.

**Table 4:** Anthropomorphic phantoms end‐to‐end testing for the Proteus^®^ONE system

Phantom	TLD			Gamma			
	Location	IROC vs. WK	Criteria	Film plane	Gamma criteria	Gamma index	RPC criteria
Anthropomorphic pelvic prostate)	Center prostate (L)	1.00	0.89–1.03	Coronal	Dose = 3%	100%	≥85%
	Center prostate (R)	1.01	0.89–1.03	Sagittal	DTA = 3 mm	99%	≥85%
Anthropomorphic head	Target TLD (L)	0.97	0.95–1.05	Coronal	Dose = 5%	86%	≥85%
	Target TLD (R)	0.97	0.95–1.05	Sagittal	DTA = 3 mm	98%	≥85%
Anthropomorphic lung	Target superior	0.92	0.92–1.05	Axial	Dose = 7%	87%	≥80%
	Target inferior	0.92	0.92–1.05	Coronal	DTA = 5 mm	92%	≥80%
				Sagittal		86%	≥80%
				Avg		88%	≥80%

### 3.E Variable virtual SAD (VSAD) measurement

Evaluation of Lynx measurements indicated that the VSAD ranged between 7.5 and 12 m near the field boundaries along the direction of the bending magnet (*y*‐axis). The VSAD at isocenter was determined to be 8.8 m. A model with a linear variable VSAD as a function of beam deflection in the y‐direction was incorporated into RayStation and used to compare dose differences between clinical plans with and without the effects of variable VSAD. Dose differences were most pronounced for plans with deep ranges and large beam deflections along the bending magnet direction. However, the maximum observed difference was less than 2.5% per total treatment and was observed at shallow depths (i.e., in skin and superficial tissues outside the treatment volume) near field boundaries along the bending magnet direction. It was determined that dosimetric impact of Proteus^®^ONE variable VSAD was negligible and that clinical use of a constant VSAD in the TPS model was appropriate.

### 3.F Image guidance system commissioning

For all three modes of image guidance, commissioning measurements validated that the modality was performing within the manufacturer's acceptable criteria (results not shown).

Film test results indicate that the distance between the proton beam axis and the oblique imaging system axis is less than 1 mm. Similarly, results from XRV100 measurements show that the proton beam axis and the oblique image system axis are coincident within 1 mm for all gantry angles (data not shown); potentially making this device a compliment to gantry star shot QA. The same set up was also used to verify CBCT imaging versus radiation isocenter coincidence (results not shown).

## 4 DISCUSSION

The Proteus^®^ONE system has many advantageuous aspects. Robust safety interlocks help to avoid harm to the patient during or as a result of treatment, such as the maximum MU per spot limitation that is crucial for patient safety. Other advantages include the Proteus^®^ONE's inherent dose delivery stability as daily QA measurements have shown an output and spot position variation of less than 2% over 1 year. Yet another is system reliability with machine up‐time over the first 2 years having been 97.7%. However, as with any system, there are areas of limitation and concern.

A workflow limitation of the current IBA Proteus^®^ONE system is that patient setup images are not managed by the OIS for acquisition of either “Setup” or “Port” films before beam delivery. Instead, they are managed by the adaPTinsight^TM^ application, restricting the ability of both the physicist and physician to perform remote review of the images.

During the measurement process of integrated depth doses, there was initially some concern regarding incomplete charge collection by the 8 cm diameter PTW Bragg peak chamber.^1^, ^2^ Saini et al. 2 presented a quantitation of charge loss during IDD measurements for their system but noted TPS performance was clinically acceptable under most conditions. Given the smaller in‐air spot sigma of our system, we expected and observed similar results. With the exception of specific instances involving a range shifter discussed below, our TPS has performed as expected during all validation tests.

The calibration curve of Hounsfield units to physical density was tested against five patient CT scans as discussed previously. The variation observed in mean physical density in adipose, muscle, and cortical bone was less than 3% compared to ICRU 49 values as demonstrated in Table 2. As detailed previously, relative linear stopping power (RLSP) comparisons were performed for the five patient scans and tissues as well as the Gammex tissue characterization plugs. RayStation compared well against both the independent stoichiometric calibration, the method presented by Fippel and Soukup,^15^ and, where applicable, ICRU 49. Differences in RayStation RLSP for the five patients did not exceed 2% compared with the two calculation methods and did not exceed 2.5% compared with ICRU 49. Differences in RayStation's RLSP for the Gammex plugs did not exceed 2.5% compared to any value, with the exception of the “Lung 300” material. In that case, RayStation overestimated the RLSP compared to the stoichiometric calibration by 4.7% but showed no deviation compared to Fippel and Soukup.^15^ As both RayStation and Fippel and Soukup utilize physical density based conversions to stopping power, this disagreement could indicate a need for future improvement with these methods. Ultimately, any concern with lung dose calculations performed by RayStation was minimized after our institution successfully met all criteria of the IROC anthropomorphic proton lung phantom evaluation (see below/Table 4).

Independent assessments of our entire proton treatment process were performed prior to the treatment of the first patient. Our process met all criteria of the IROC absolute dose TLD checks and passed a comprehensive review by a group of proton physicists. Within 6 months, our facility successfully met all criteria of the IROC anthropomorphic prostate, head and lung phantom tests, as demonstrated in Table 4.

We note several areas where further development of either the Proteus^®^ONE system or the RayStation TPS are warranted. In particular, for plans with range shifters, there is no explicit modeling of the nuclear halo^13^, ^16^ broadening in the air gap in between the range shifter and the patient. The RayStation manual states that “the pencil‐beam dose algorithm only takes the extra widening of the multiple Coulomb Scattering (MCS) part of the beam into account. Thus, increases in the penumbrae and the removal of dose from the central part of the beam is not accurately modeled”.^14^ While this is not typically an issue regarding inhomogeneities in the patient, it does become problematic for shallow treatment fields when there is a large air gap resulting in TPS doses that are inaccurately high. Consequently, a robust QA process has been implemented at Willis‐Knighton Cancer Center to evaluate such dose disagreements between the TPS and measurement. Furthermore, WKCC takes care to reduce the air gap between patient surface and range shifter for targets of less than 4‐cm depth, as preliminary data indicate that a 10‐cm air gap yields 2% disagreement between TPS and measurement within the proximal portion of the SOBP.

## 5 CONCLUSION

The first commercial, clinically utilized, compact, image‐guided, pencil‐beam scanning, intensity‐modulated proton therapy system, the IBA Proteus^®^ONE, was installed at the Willis‐Knighton Cancer Center (Shreveport, LA) in 2014. Major tasks associated with characterization and clinical commissioning of this machine included mechanical and radiation isocenter checks, radiation safety checks, beam dose/MU calibration, beam data collection for beam modeling in the RayStation treatment planning system (TPS), baseline data for periodic quality assurance checks, TPS dose calculation accuracy validation, imaging system functionality tests, and end‐to‐end tests for simulation, planning, and dose delivery with an anthropomorphic phantom. All of these tasks were successfully completed with results summarized in this paper. The WKCC Proteus^®^ONE machine was released for clinical use in September 2014 and a robust quality assurance program has been implemented to ensure safe and accurate proton therapy dose delivery to our patients. Over three hundred and thirty patients, including pediatric, with various treatment sites such prostate, breast, lung, head and neck, whole pelvis, abdomen, and brain have been treated at our center to date. In all, the Proteus^®^ONE has been found to be stable and reliable, delivering planned dose distributions to the patient's target volume within established tolerance limits.

## CONFLICT OF INTEREST

No conflict of interest.
